# Drug resistance mechanisms in cancers: Execution of pro-survival strategies

**DOI:** 10.7555/JBR.37.20230248

**Published:** 2024-02-28

**Authors:** Pavan Kumar Dhanyamraju

**Affiliations:** Fels Cancer Institute of Personalized Medicine, Lewis-Katz School of Medicine, Temple University, Philadelphia, PA 19140, USA

**Keywords:** cancer, drug resistance, mechanisms, microRNAs, treatment strategies

## Abstract

One of the quintessential challenges in cancer treatment is drug resistance. Several mechanisms of drug resistance have been described to date, and new modes of drug resistance continue to be discovered. The phenomenon of cancer drug resistance is now widespread, with approximately 90% of cancer-related deaths associated with drug resistance. Despite significant advances in the drug discovery process, the emergence of innate and acquired mechanisms of drug resistance has impeded the progress in cancer therapy. Therefore, understanding the mechanisms of drug resistance and the various pathways involved is integral to treatment modalities. In the present review, I discuss the different mechanisms of drug resistance in cancer cells, including DNA damage repair, epithelial to mesenchymal transition, inhibition of cell death, alteration of drug targets, inactivation of drugs, deregulation of cellular energetics, immune evasion, tumor-promoting inflammation, genome instability, and other contributing epigenetic factors. Furthermore, I highlight available treatment options and conclude with future directions.

## Introduction

Cancer is a highly complex heterogeneous disease characterized by uncontrolled cell proliferation and dynamic genomic alterations^[1]^. It is one of the most dreadful diseases of the 21st century and is the leading cause of death worldwide^[2]^. According to the American Cancer Society, approximately 1958310 new cancer cases are expected to be diagnosed in 2023, with an estimated 609820 deaths^[3]^. To understand the distinguishing features of cancer cells, Hanahan and Weinberg in 2000 identified the hallmarks of cancer cells, which include self-sufficiency in growth signals, insensitivity to anti-growth signals, tissue invasion and metastasis, limitless replicative potential, sustained angiogenesis, and evading apoptosis^[4]^. In 2011, the same authors^[5]^ added four additional characteristics of cancer cells, which include avoiding immune destruction, deregulating cellular energetics, tumor-promoting inflammation, genome instability, and mutation^[5]^. Alterations in normal cells because of genetic and/or environmental factors, leading to the acquisition of these hallmark characteristics, can lead to malignant transformation and ultimately full-blown cancer. Importantly, these hallmarks illustrate how various cellular, physiological, and immunological aspects are affected in a cancerous cell^[6]^. Thus, an in-depth understanding of these characteristics is indispensable for developing treatment modalities. For cancer patients, currently available therapeutic interventions comprise surgery, radiation therapy, cytotoxic chemotherapy, immunotherapy, endocrine therapy, and targeted therapy^[7]^.

Despite significant progress in cancer therapeutics, drug resistance continues to be one of the major hurdles in cancer treatment. In the present review, I examine the phenomenon of drug resistance and describe various types of drug resistance mechanisms, including DNA damage repair, inhibition of cell death, alteration of drug target, inactivation of the drug, epigenetics, epithelial to mesenchymal transition, drug efflux, deregulation of cellular energetics, immune evasion, tumor-promoting inflammation, and genome instability. Additionally, I discuss treatment options and conclude with future perspectives.

## Drug resistance

**Figure 1:**
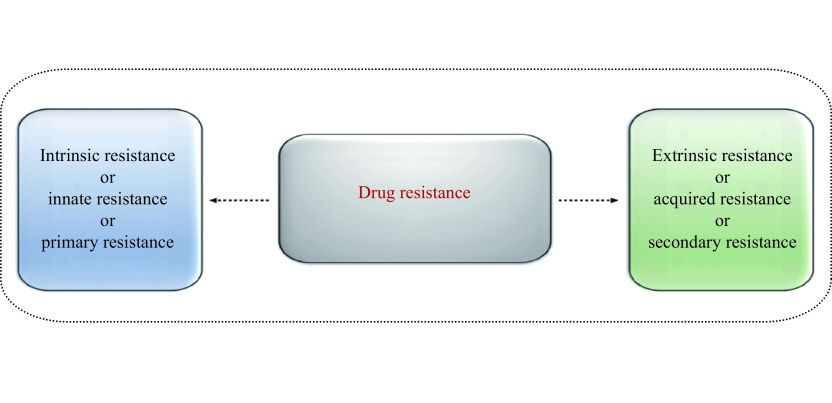
Modes of drug resistance.

Cells insensitive to or tolerant of a therapeutic agent are considered drug resistant. Drug resistance was initially observed in infections of certain bacteria that developed resistance to antibiotic treatment^[8]^. Later, identical mechanisms were observed in cancer cells, making drug resistance the Achilles' heel of cancer therapeutics^[9]^. Although the concept of drug resistance is not new, significant progress in understanding the underlying molecular and cellular mechanisms of drug resistance over the past two decades has emphasized its importance and brought it back to the scientific spotlight. Recently, inefficient drug distribution and pharmacokinetic reasons for drug resistance have also been put forward^[10]^. In principle, cancer drug resistance can be categorized into two types: (a) intrinsic/innate/primary resistance and (b) extrinsic/acquired/secondary resistance, as shown in ***Fig. 1***. Intrinsic/innate/primary resistance, as the name suggests, is the inherent ability of cancer cells to resist a drug even before the administration of the drug^[11]^. An inadequate response to the drug by the patient suggests the existence of innate resistance against the drug. Conversely, extrinsic/acquired/secondary resistance develops when cancer cells are initially susceptible to a drug but later become resistant because of alterations in proteins and/or genetic perturbations^[11]^. Additionally, treating acquired resistance poses a greater challenge, compared with innate resistance. Multiple mechanisms of drug resistance have been identified and will be discussed one by one.

## Mechanisms of drug resistance

### Drug efflux

**Figure 2:**
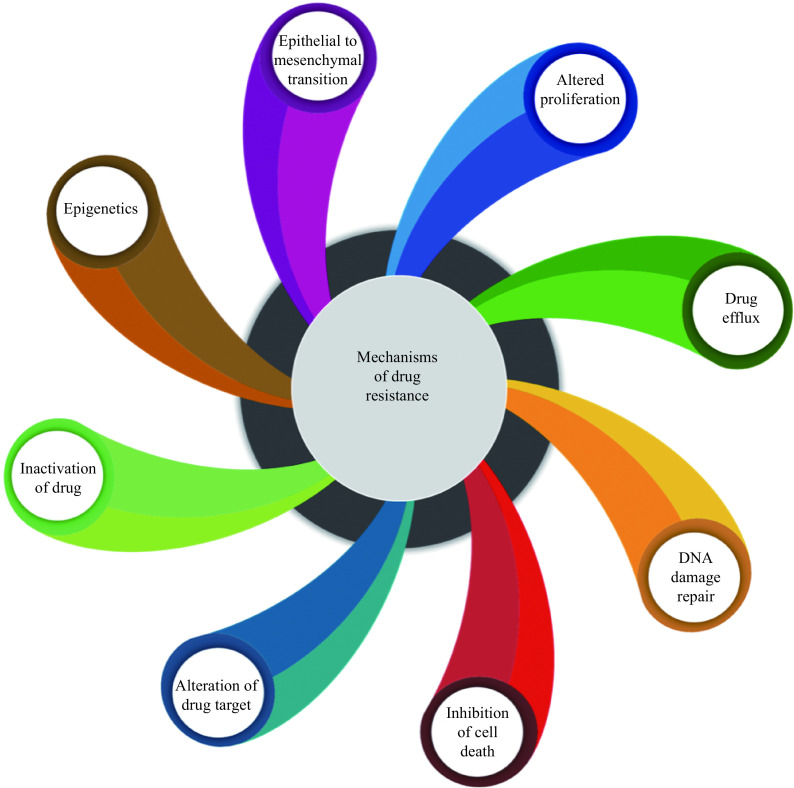
A graphical illustration of drug resistance mechanisms in cancer cells.

Among various mechanisms of drug resistance depicted in ***Table 1*** and ***Fig. 2***, drug efflux is an extensively studied and well-understood mechanism. In simple terms, "efflux" refers to the expulsion of diverse substrates out of the cells. It is primarily mediated through energy-dependent transmembrane transport proteins, known as ATP-binding cassette (ABC) proteins^[12]^. These proteins are present on the plasma membrane of cells and have been shown to play various key functions, including the active pumping of drugs out of cells and the regulation of ions, lipids, hormones, and xenobiotics at the cellular level. The roles of these proteins in the maintenance of the blood-brain barrier have also been established^[12]^. Furthermore, these proteins also play other crucial physiological roles, such as the regulation of intracellular organelles, including lysosomes, mitochondria, the Golgi apparatus, and the endoplasmic reticulum^[12]^. Given the multi-functionality of these proteins, any perturbation in the coding genes can lead to rare genetic disorders, such as cystic fibrosis, Tangier disease, and several others^[12]^.

**Table 1:** Various modes of drug resistance mechanisms and their characteristics

Drug resistance mechanisms	Genes/proteins/pathways affected	Resistance against drugs	References
Drug efflux	High expression of ABC transporters: P-gp, BCRP, and MRP	Doxorubicin, topotecan, gefitinib, sorafenib, axitinib, flavopiridol, methotrexate, dasatinib, canertinib, imatinib, bisantrene, erlotinib hydrochloride, nilotinib, and sunitinib malate	[20]
DNA damage repair	High expression of MGMT	Temozolomide	[43]
	Phosphorylation (pY240-PTEN)	Radioresistance	[44]
	Loss of APC	Doxorubicin	[45]
	Downregulation of ER protein and DNA repair machinery, upregulation of IL-6/STAT3 pathway	Palbociclib	[46]
	Elevated levels of DNA repair capacity genes	Cisplatin	[47,188–189]
	High expression levels of BRCA1, BRCA2, RPA1, and Rad51	Radioresistance	[48]
Inhibition of cell death	Mutations in anti-apoptotic genes: BCL-2 family, caspases, IAPs, MCL-1	Gemcitabine and rituximab	[56]
	Overexpression of genes (*ABCB1* and *ALDH1A1*)	Carboplatin, temozolomide, taxol VP16, and LCL161,	[89–92,94]
Alteration of drug target	Beta-tubulin mutations, DNA topoisomerase Ⅱ	Paclitaxel and doxorubicin	[57]
	Reactivation of BCR-ABL signaling	STI-571 (Abl tyrosine kinase inhibitor)	[58]
	EGFR mutations	Gefitinib	[61]
	Genomic amplification of androgen receptor	Bicalutamide and leuprolide	[190–191]
Inactivation of drugs	Decreased drug activation because of mutations or downregulation of deoxycytidine kinase	Cytarabine	[66]
	Mutations in CYP2B6	Cyclophosphamide	[71]
	Mutations in CYP2D6	Tamoxifen	[71]
	High UGT expression	Anthracycline, daunorubicin	[74–76]
	Elevated levels of UGT1A	Luminespib and ganetespib	[77]
Epigenetics	Overexpression of SETD5	MEK inhibitors	[84]
	Downregulation of glutathione metabolism and cytochrome p450 pathway	MEK inhibitors	[85]
	Elevated ABCB1 levels	Adriamycin	[86]
	Reduced GAS5 levels	Adriamycin	[86]
	Upregulation of UCA1	Cisplatin	[87]
	AKAP12 hypomethylation	Paclitaxel	[89]
	Hypomethylation of CpG sites on S100A4	Cisplatin	[90]
	Overexpression of BMP4	Cisplatin	[91]

**Table 1:** Various modes of drug resistance mechanisms and their characteristics (continued)

Drug resistance mechanisms	Genes/proteins/pathways affected	Resistance against drugs	References
Epithelial-to-mesenchymal transition	Upregulation of breast cancer stem cell genes (*TGF-β*, *ALDH1A1*, *CD44*, and *JUN*)	Docetaxel, paclitaxel and anthracycline	[101–102]
	Upregulation of PARP1, TWIST1, and SNAIL	Doxorubicin	[147,192]
Deregulation of cellular energetics	Decreased PK-M2 expression	Cisplatin	[110]
	Increased HK2 expression	Tamoxifen	[114]
Immune evasion/avoiding immune destruction	Synthesis of indoleamine 2,3-dioxygenase promotes T-cell suppression	Anti-CTLA-4 and anti-PD-1 blocking antibodies	[131]
	Loss of PTEN in tumor cells	Worse PD-1 inhibitor response	[133]
Tumor-promoting inflammation	Upregulation of CXCR7	Cisplatin	[138]
	Activation of STAT3	Tyrosine kinase inhibitors	[139–140]
	Increased TGF-beta-dependent IL-6 secretion	Erlotinib	[142]
	Overexpression of P-gp	Cisplatin	[144]
	Overexpression of IRAK4	Gemcitabine	[145]
	Overexpression of TGF-β	Sorafenib	[146]
Genome instability and mutations	Aneuploidy	Paclitaxel and cisplatin	[150]
	Chromosomal instability	Carboplatin and paclitaxel	[152]
Abbreviations: ABC, ATP-binding cassette; ABCB1, ATP-binding cassette sub-family B member 1; ALDH1A1, aldehyde dehydrogenase 1 family member A1; APC, adenomatous polyposis coli; AKAP12, A-kinase anchor protein 12; BCRP, breast cancer resistance protein; BCL-2, B-cell lymphoma 2; BMP4, bone morphogenetic protein 4; BRCA1, breast cancer gene 1; CD44, type I transmembrane protein; CTLA-4, cytotoxic T-lymphocyte-associated protein 4; CXCR7, C-X-C motif chemokine receptor 7; CYP2B6, cytochrome P450 2B6; CYP2D6, cytochrome P450 2D6; EGFR, epidermal growth factor receptor; ER, endoplasmic reticulum; GAS5, growth arrest-specific 5; HK2, hexokinase 2; IAPs, inhibitors of apoptosis proteins; IL6, interleukin 6; IRAK4, IL-1 receptor-associated kinase 4; JUN, Jun proto-oncogene; MGMT, methylguanine DNA methyltransferase; MRP, multidrug resistance-associated protein; PD-1, programmed cell death protein 1; P-gp, permeability glycoprotein; PK-M2, pyruvate kinase M2; PTEN, phosphate and tensin homolog; RPA1, replication protein A1; RAD51, recombinase Rad51; STAT3, signal transducer and activator of transcription 3; SETD5, SET domain containing 5; TGF-β, transforming growth factor beta; UCA1, urothelial cancer-associated 1; UGT1A, UDP glucuronosyltransferase 1 family, polypeptide A cluster.			

One of the fundamental reasons for cancer chemoresistance is attributed to the enhanced activities of these transporters, which leads to an overall reduction in intracellular concentration of the drugs, ultimately resulting in insensitivity to chemotherapeutic agents^[13]^. In humans, approximately 48 members of ABC transporters have been identified and categorized into seven sub-families including ABCA, ABCB, ABCC, ABCD, ABCE, ABCF, and ABCG^[14]^. These proteins are expressed in all tissues, but intestinal and liver epithelial cells have been shown to have the highest expression of ABC transport proteins^[12,15]^. These proteins can be identified by two unique domains: the nucleotide-binding domain (NBD) and the transmembrane domain (TMD)^[16–17]^. The nucleotide-binding domain is highly conserved, while the transmembrane domain is variable. Upon substrate binding to the transmembrane domain, hydrolysis of adenosine triphosphate (ATP) takes place at the nucleotide-binding site, causing a conformational change that leads to the expulsion of the substrate out of the cell^[18]^.

Among ABC transport proteins, the well-characterized proteins that are engaged in drug resistance are breast cancer resistance protein (BCRP), multidrug resistance-associated protein (MRP), and permeability glycoprotein (P-gp). The 72-kDa protein BCRP is coded by the *ABCG2* gene located on chromosome 4q22^[19]^. It belongs to the G-subfamily of ABC transporters and demonstrates a wide inhibitor and substrate specificity^[12]^. BCRP is expressed in stem cells and plays a crucial role in folate and heme homeostasis^[20]^. It renders cells resistant to a myriad of anti-cancer agents. Multiple studies have demonstrated that the overexpression of BCRP leads to drug resistance in cells^[21]^.

For instance, one study has shown BCRP to be overexpressed in breast cancer cell line MCF7 that is resistant to doxorubicin^[22]^ (***Table 2***), suggesting BCRP confers resistance against doxorubicin in the MCF7 cell line^[22]^. In chronic myeloid leukemia (CML) patients, high BCRP expression was noticed in early CML cell populations and CD34^+^ cells^[23]^. Ross *et al*^[24]^ demonstrated a high expression level of BCRP in samples from acute myeloid leukemia (AML) patients, indicating its role in imparting resistance to traditional chemotherapeutics. In another study, Kawabata *et al*^[25]^ observed high *BCRP* mRNA levels in non-small cell lung cancer (NSCLC) cell lines that are correlated with the efflux of topotecan. The BCRP efflux pump acts against a broad range of xenobiotics, including gefitinib, sorafenib, axitinib, flavopiridol, methotrexate, dasatinib, canertinib, imatinib, bisantrene, erlotinib hydrochloride, nilotinib, and sunitinib malate, thus categorizing BCRP as a multidrug resistance (MDR) transporter^[20]^.

**Table 2:** Drugs approved by the United States Food and Drug Administration for treating various cancers

Drugs	Trade names	Manufacturer/company	References
Axitinib	Inlyta	Pfizer	[20]
Bisantrene	Zantrene	Race Oncology	[20]
Doxorubicin	Doxil	Baxter International	[20]
Carboplatin	Paraplatin	Accord Biopharma	[152]
Capecitabine	Xeloda	Genentech	[176]
Cyclosporine	Neoral	Novartis	[20]
Cyclophosphamide	Cytoxan	Bristol-Myers Squibb	[68]
Cytarabine	Cytosar-U	Pfizer	[66]
Dacarbazine	DTIC-Dome	Hikma Pharmaceuticals USA Inc	[68]
Dasatinib	Sprycel	Bristol-Myers Squibb	[20]
Daunorubicin	Daunotec	Cipla	[20]
Docetaxel	Taxotere	Sanofi-Aventis US LLC	[20]
Etoposide	Toposar	Pfizer	[20]
Erlotinib hydrochloride	Tarceva	Roche	[20]
Epirubicin	Ellence	Pfizer	[20]
Flavopiridol	Alvocidib	Tolero Pharmaceuticals	[20]
Gefitinib	Gilotrif	Boehringer Ingelheim	[20]
Ifosfamide	Ifex	Baxter Healthcare Corporation	[68]

**Table 2:** Drugs approved by the United States Food and Drug Administration for treating various cancers (continued)

Drugs	Trade names	Manufacturer/company	References
Imatinib	Gleevec	Novartis	[20]
Ipilimumab	Yervoy	Bristol Myers Squibb	[131]
Methotrexate	Trexall	Teva Czech Industries	[20]
Nilotinib	Tasigna	Novartis	[20]
Oxaliplatin	Eloxatin	Sanofi	[164]
Paclitaxel	Paclisal	Salvavidas Pharma	[20]
Palbociclib	Ibrance	Pfizer	[46]
Procarbazine	Matulane	Leadiant Biosciences	[68]
Sorafenib	Nexavar	Bayer HealthCare Pharmaceuticals	[20]
Sunitinib malate	Sutent	Pfizer	[20]
Tamoxifen	Nolvadex	AstraZeneca AB	[20]
Temozolomide	Temonot	Natco	[43]
Thiotepa	Tepadina	Amneal Pharmaceuticals	[68]
Topotecan	Hycamtin	Novartis	[20]
Vinblastine	Velban	Cipla	[20]
Vincristine	Cytocristin	Cipla	[20]

BCRP was isolated from mitoxantrone-resistant human colon cancer cells, hence it was also named mitoxantrone resistance protein (MXR)^[26]^. It was demonstrated that BCRP actively effluxes mitoxantrone from the cells, thereby reducing its intracellular accumulation^[20]^. Apart from mitoxantrone-resistant colon cancer cells, BCRP has also been observed to be overexpressed in a myriad of mitoxantrone-resistant cancer cell lines, including myeloma, fibrosarcoma, glioblastoma, and gastric carcinoma^[20,27]^. Considering the plethora of functions executed by BCRP, it has become a key pharmacological target to overcome drug resistance in cancer patients.

In a study conducted in 1992, Cole *et al*^[28]^ identified the MRP. The MRP subfamily of proteins belongs to the C-subset of the ABC transporter superfamily and consists of nine members^[29]^. The human MRP family includes the MRP proteins (MRP1 to MRP9, gene symbols *ABCC1* to *ABCC6* and *ABCC10* to *ABCC12*), the cystic fibrosis transmembrane conductance regulator (ABCC7) protein, and the sulfonylurea receptor (SUR) proteins (SUR1 and SUR2, gene symbols *ABCC8* and *ABCC9*)^[30]^. These MRP proteins are capable of transporting a wide range of xenobiotics and chemotherapeutic agents, playing a vital role in drug absorption, disposition, and efflux of these compounds from cells. Moreover, elevated expression levels of MRPs have been associated with a myriad of cancers, including NSCLC, leukemias, esophageal, breast, kidney, ovary, and colon cancers, and are associated with poor clinical outcomes^[31–32]^.

For example, MRP1 has been demonstrated to contribute to resistance against several drugs, including etoposide, doxorubicin, daunorubicin, vincristine, and methotrexate; additionally, its expression levels have been reported to be high in NSCLs, leukemias, and esophageal cancers. Increased expression of MRP2 has been shown in AML and cancers of the breast, lung, kidney, ovary, colon, and liver. Furthermore, MRP2 is known to offer resistance against drugs including cisplatin, vincristine, paclitaxel, and methotrexate. Ovarian cancer, acute lymphoblastic leukemia (ALL), and hepatocellular cancer have all been found to have elevated MRP3 expression levels. Human epirubicin-resistant leukemic cells have also been reported to have high MRP6 levels, while non-small cell lung cancer cells are known to express elevated levels of MRP7^[33]^.

Yamada *et al*^[34]^ conducted a study using a tissue microarray with 281 breast cancer patient samples (17 luminal A, 27 human epidermal growth factor receptor 2 [HER2], 46 triple-negative, and 191 luminal A breast cancer) to investigate the expression levels of various ABC transporters; the expression levels of ABCC1 and ABCC11 were found to be associated with a substantially shorter disease-free survival. In aggressive subtypes, ABCG2, ABCC1, and ABCC11 were expressed more often and at substantially higher levels, compared with ABCB1, and patients with HER2-positive and triple-negative tumors expressed significantly higher levels of ABCC11 and had a poorer disease-free survival^[34]^.

Another important member of the ABC transporters, which has been extensively studied, is the P-gp that is a dimeric glycosylated 170-kDa protein product of the *MDR1* (*ABCB1*) gene. P-gp comprises two six-membered TMDs and two homologous NBDs. The TMD makes an extracellular conduit for P-gp substrates, while the NBD by using ATP catalyzes the export of substrates from the cell. P-gp protects the cell against toxic and xenobiotic compounds, and P-gp is implicated in the modulation of endogenous material transport^[35]^. P-gp is found in a myriad of cell types, including bone marrow stem cells and cells of the brain, kidney, testis, liver, gut, placenta, adrenal glands, large intestine, and other tissues^[36]^. The upregulation of P-gp has been observed in response to a variety of chemotherapeutic agents, which results in the efflux of the drugs, ultimately leading to decreased intracellular concentrations and thereby curbing their therapeutic potential. However, the exact mechanism through which P-gp functions remains enigmatic. Cancer patients undergoing doxorubicin treatment demonstrated an upregulation of P-gp that had a very broad substrate spectrum, allowing it to mediate the export of a plethora of anticancer agents, including docetaxel, vinblastine, paclitaxel, lovastatin, atorvastatin, etoposide, vincristine, daunorubicin, digitoxin, digoxin, cyclosporine, and gefitinib, among many others^[37]^. In 2019, He *et al*^[38]^ demonstrated that the expression levels of P-gp were correlated with cisplatin efficacy in osteosarcoma patients; sh-RNA-based molecular knockdown of P-gp led to sensitization of osteosarcoma cells to cisplatin; furthermore, knocking down of P-gp in osteosarcoma was shown to be a potential approach for overcoming cisplatin resistance; and the extensive role of drug efflux mechanisms and their components in cancer drug resistance make them potential targets for therapeutic interventions.

### DNA damage repair

DNA damage has long been acknowledged as a contributing element to the genesis of cancer. DNA repair pathways in mammals are primarily involved in the maintenance of genomic integrity^[39]^. Exposure of normal cells to genotoxic agents, including physical agents such as ionizing radiations (IRs), ultraviolet (UV) light, thermal disruption, and/or chemical agents, leads to the generation of DNA adducts. These adducts are generated *via* chemical modifications to sugars or bases, single-strand breaks (SSBs), or double-strand breaks (DSBs). Furthermore, unrepaired DNA lesions eventually lead to the formation of mutations that cause the initiation as well as progression of cancer^[39]^. If any oncogenes or tumor suppressor genes are disrupted by these lesions, the likelihood of developing cancer increases significantly^[40]^. Multiple research reports link defects in repair pathways to the development of specific types of cancer. For instance, 70% of cases of hereditary nonpolyposis colorectal cancer (HNPCC) are caused by germline mutations in mismatch repair (MMR) genes. Another example is the *BRCA1* and *BRCA2* genes that are critically involved in the DNA repair pathway^[39]^, and germline mutations in these genes confer genetic predisposition to cancers of the ovary, breast, and pancreas^[41]^. Moreover, the development of resistance to myriad cancer therapies, including radiation therapy, chemotherapy, and immunotherapy, has been a major stumbling block in treating cancers^[42]^. Therefore, targeting the DNA repair pathways has become a primary focus of several research investigations.

For example, glioblastoma multiforme (GBM) is a severe brain cancer with very low survival rates. The primary treatments include temozolomide (TMZ)-containing chemotherapy, radiotherapy, and surgical resection. The use of TMZ leads to the generation of O^6^-methylguanine adducts, resulting in DSB post-replication. There is evidence that the DNA damage repair enzyme 6-methylguanine DNA methyltransferase (MGMT) contributes to TMZ resistance. The DNA repair protein MGMT favors damage repair over DSB production and cell death. Furthermore, it removes the O^6^-methylguanine adducts. In this manner, MGMT inhibitors suppress MGMT activity^[43]^.

In a study conducted in 2019, Ma *et al*^[44]^ demonstrated that exposure of glioblastoma cells to ionizing radiation (IR) induces phosphate and tensin homolog (PTEN) Y240 phosphorylation (pY240-PTEN), mediated by fibroblast growth factor receptor 2 (FGFR2). Phosphorylation of PTEN leads to its binding to chromatin, thereby recruiting RAD51 that promotes DNA repair. Furthermore, inhibition of Y240 phosphorylation sensitizes GBM to IR and prolongs the survival of GBM in preclinical models. These findings imply that pY240-PTEN, regulated by FGFR, is a fundamental mechanism of radiation resistance and a therapeutic target for increasing radiotherapy efficacy^[44]^.

The tumor suppressor gene adenomatous polyposis coli (*APC*) has been shown to be lost in approximately 70% of sporadic breast cancers through hypermethylation or *via* mutations. In 2019, Stefanski *et al*^[45]^ demonstrated that after 24 h of treatment with doxorubicin, MMTV-PyMT;*Apc*^Min/+^ cells had less DNA damage compared with MMTV-PyMT;*Apc*^+/+^ cells; additionally, a reduction in DNA damage response pathways, including ataxia telangiectasia mutated (ATM), checkpoint kinase 1 (CHK1), and CHK2, was reported at 24 h in MMTVPyMT;*Apc*^Min/+^ cells compared with the control cells; finally, the authors showed that the use of inhibitors targeting DNA damage repair kinases (ATR, DNAPK, and ATM) increased the amount of doxorubicin-induced apoptosis in the MMTVPyMT;*Apc*^Min/+^ cells.

In 2019, Kettner *et al*^[46]^ demonstrated that estrogen receptor-positive breast cancer cells develop resistance to the CDK4/6 inhibitor palbociclib by downregulating ER protein and DNA repair machinery and upregulating the IL-6/signal transducer and activator of transcription 3 (STAT3) pathway. It was observed that when matched biopsies from breast cancer patients who developed the disease while taking palbociclib and the samples from their initial biopsies (before treatment with palbociclib), it was uncovered that DNA repair, ER, and IL-6/STAT3 were dysregulated; therefore, the authors proposed that this resistance might be overcome by using PARP and STAT3 inhibitors^[46]^.

In another study, it was shown that tumor cells treated with cisplatin were initially sensitive in a mouse model of human lung cancer, but with continued treatment, resistance developed; upon further analysis, high expression levels of genes related to DNA damage repair and DNA repair capacity (DRC) were found in cisplatin-resistant tumor cells; additionally, inhibition of the NER pathway in these tumor cells led to increased susceptibility to cisplatin^[47]^.

Liu *et al*^[48]^ established the FaDu-RR hypopharyngeal cancer cell line by repeatedly subjecting the FaDu cell line to ionizing radiation, with a cumulative dosage of 60 Gy; additionally, they carried out a microarray and bioinformatics study of FaDu-RR cells and discovered that four essential proteins in homologous recombination pathways, BRCA1, BRCA2, RPA1, and RAD51, express themselves at significantly higher levels in FaDu-RR cells than in the control cells; furthermore, silencing RPA1 reduced the radioresistance of FaDu-RR cells. Therefore, considering the major role played by DNA repair mechanisms and the genes involved in these mechanisms, targeting them would be beneficial in treating diverse cancers.

### Inhibition of cell death (blockade of apoptosis)

One of the fundamental strategies executed by cancer cells to resist chemotherapy and radiotherapy is *via* the inhibition of cell death (avoiding/resisting apoptosis)^[49]^. Cancer cells are genomically unstable, resulting in alterations such as gene amplification, gene overexpression, and mutations in anti-apoptotic genes, such as the B-cell lymphoma 2 (BCL-2) family, caspases, and inhibitors of apoptosis proteins (IAPs), all of which contribute to treatment resistance^[50]^. Apoptosis is a highly sophisticated and regulated process fundamentally required for genome integrity, the proper functioning of the immune system, normal embryonic development, and the maintenance of tissue homeostasis. Two independent signaling pathways are associated with apoptosis: (a) the intrinsic pathway and (b) the extrinsic pathway^[51]^. The intrinsic pathway is primarily mediated by mitochondria and involves caspase-9, AKT, and the BCL-2 protein family, whereas the extrinsic pathway involves death receptors on the cell surface. The intrinsic, as well as extrinsic processes, converge upon downstream activation of caspase-3, resulting in apoptosis.

Using fluorescence-activated cell sorting, Liu *et al*^[52]^ determined the proportion of CD133^+^ cells in three separate primary cultured cell lines from glioblastoma patients. They showed that CD133^+^ cells were more resistant to chemotherapeutic agents, such as carboplatin, temozolomide, taxol, and VP16 than CD133^−^ cells; furthermore, CD133^+^ cells had higher expression levels of BCL-XL, BCL-2, FLIP, and many IAPs, which include neuronal apoptosis inhibitory protein (NAIP), X-linked lymphoproliferative syndrome type 2 (XIAP), cIAP1, cIAP2, and survivin, than the CD133^−^ cells^[52]^. Their study, for the first time, provided evidence that CD133^+^ cells contribute to chemoresistance, which in turn was brought on by higher expression of anti-apoptosis protein and inhibitors of apoptosis protein families^[52]^.

In a study conducted in 2012, Chen *et al*^[53]^ used a second mitochondrial-derived activator of caspases (SMAC) mimetic, LCL161, on four different hepatocellular carcinoma (HCC) cell lines, namely PLC5, Hep3B, Huh-7, and SK-Hep1, and found that LCL161 showed differential effects on apoptosis in these cells; furthermore, the sensitivity of HCC cells to LCL161 depended on the endogenous levels of BCL-2; sensitive Hep3B and PLC5 cells, expressing lower levels of BCL-2, undergo cytotoxicity and apoptosis, whereas resistant SK-Hep1 and Huh-7 cells, expressing higher levels of BCL-2, do not. It was shown that the resistance to LCL161 was overcome in Huh-7 by BCL-2 downregulation through short interference RNA, and the anti-apoptotic effect was restored in BCL-2-expressing Hep3 cells. These results suggest that BCL-2 contributes to chemoresistance and targeting BCL-2 may overcome resistance to LCL161. Another member of the BCL-2 family of proteins, myeloid cell leukemia-1 (MCL-1), also has anti-apoptotic properties. In several malignancies, including cancers of the CNS, colon, breast, lung, ovary, kidney, and prostate as well as melanoma, MCL-1 has been shown to be highly expressed^[54]^.

Wei *et al*^[55]^ stably expressed shRNAs targeting the *MCL-1* gene in pancreatic cancer cell lines, namely SW1900, BxPC-3, and PANC-1, and showed that downregulation of MCL-1 caused apoptosis, inhibited cell growth, cell cycle arrest, and colony formation in pancreatic cell lines; additionally, MCL-1 knockdown led to an increased sensitivity to the standard of care drug gemcitabine in pancreatic cell lines.

Hussain *et al*^[56]^ conducted a study in 2007 and demonstrated that siRNA-mediated MCL-1 knockdown resulted in mitochondrial membrane depolarization and apoptosis in ALL cell lines and tumor cells from chronic lymphocytic leukemia (CLL) patients; furthermore, MCL-1 down-regulation led to enhanced sensitivity to rituximab-mediated killing by complement-dependent cytotoxicity and direct apoptosis. In conclusion, MCL-1 is a therapeutic target for treating CLL and ALL, and its down-regulation may improve the therapeutic efficacy.

Taken together, cancer cells avoid apoptosis through multiple ways and continue to grow and proliferate. Therefore, understanding the role of apoptosis in cancer biology and tackling the evasion of apoptosis by cancer cells should be considered for future treatments.

### Alteration of drug target

The binding and therapeutic value of a drug depends on its molecular target, and any perturbations to the target or its expression level would render the drug nonfunctional or ineffective. Target alterations are frequently observed in many types of cancers^[57]^. For example, Gorre *et al*^[58]^ in their clinical studies demonstrated that individuals with an advanced stage CML initially responded favorably to STI-571 (an ABL tyrosine kinase inhibitor) but eventually relapsed because of drug resistance; however, the reactivation of BCR-ABL signaling was identified as the primary trigger for relapse; further investigation into this revealed that the substitution of a threonine residue in the ABL kinase domain was the principal influencing factor, and substituting isoleucine for threonine was sufficient to provide STI-571 resistance.

Another well-known example of drug alteration can be found in 30% of prostate cancer patients, where the androgen receptor is genomically amplified; as a result, these patients become resistant to bicalutamide and leuprolide, which are used as androgen deprivation therapy^[57]^. Ovarian cancer cells have been found to be resistant to several taxanes, including paclitaxel, because of perturbations in therapeutic targets, such as beta-tubulin mutations, among other known mechanisms^[57]^.

DNA topoisomerase-Ⅱ inhibitors belong to the family of anti-neoplastic drugs that inhibit DNA from under coiling or supercoiling. DNA topoisomerase-Ⅱ inhibitors stabilize the transitory complex formed between DNA and topoisomerase-Ⅱ, leading to inhibition of mitosis, inhibition of DNA synthesis, and resulting in DNA damage. In many cancers, mutations in the DNA topoisomerase-Ⅱ gene are responsible for drug resistance^[59]^. Doxorubicin is a fungus-derived antibiotic that is primarily used to treat solid tumors like breast and lung cancers. The mode of action is *via* inhibiting topoisomerase-Ⅱ. Mutations in the topoisomerase-Ⅱ gene in cancer cells alter how doxorubicin works^[57]^.

Drug target alterations can be noticed because of mutations in kinases, such as the EGFR family and its downstream signaling partners, RAF, RAS, MEK, and SRC, leading to continuous activation of the signaling pathway, eventually promoting uncontrolled cell growth. In certain lung tumors with EGFR tyrosine kinase domain mutations, enhanced response rates to EGFR inhibitors are documented with acquired resistance within one year. In half of all cases, an EGFR-T790M gatekeeper mutation was observed^[60–61]^.

The above examples emphasize how cancer cells alter the drug targets, rendering the drugs nonfunctional or ineffective. Therefore, care must be taken in designing drugs that can target cancer cells in multiple ways.

### Inactivation of drugs

One of the most prevalent mechanisms of drug resistance exhibited by cancer cells is *via* genetic modification of drug targets, such as changes in expression levels and mutations^[62]^. Chemoresistance also develops because of increased drug inactivation by phase Ⅰ/Ⅱ enzymes and the reduction in the intracellular activation of prodrugs^[63]^.

Most anticancer drugs are processed predominantly in the liver, intestine, and tumor tissues by phase Ⅰ/Ⅱ enzymes. The function and efficacy of anticancer drugs rely on multiple complex processes. Drug interactions with various types of proteins *in vivo* can change the molecular features of drugs and, as a result, activate them. For example, drug inactivation has been reported in members of the GST superfamily of detoxifying enzymes, which protect cellular macromolecules against electrophilic compounds. Inhibiting the mitogen-activated protein kinase (MAPK) pathway and direct detoxification of GSTs facilitate the development of drug resistance^[64]^. Elevated GST expression levels enhance anticancer drug detoxification in cancer cells, leading to less efficient cytotoxic damage to cells. Furthermore, this increase is linked to resistance to apoptosis^[57]^.

Another example of drug resistance is through changes in apoptosis-related proteins. The tumor suppressor protein p53 promotes apoptosis in response to chemotherapy. In about 50% of tumors, p53 is mutated, rendering the gene non-functional and leading to treatment resistance. Drug resistance is also achieved by the inactivation of regulators of apoptosis, such as apoptotic protease activating factor 1 (APAF-1), caspase-9, and its cofactors^[65]^. Another well-known example is cytarabine, used in the treatment of AML^[57]^. Cytarabine is initially phosphorylated by deoxycytidine kinase to form cytarabine-monophosphate, which is further phosphorylated by nucleotide kinases, resulting in the active form, cytarabine triphosphate. Cytarabine is inactivated by the enzyme pyrimidine nucleoside deaminase, thereby converting it to a non-toxic uracil derivative. Cancer cells develop chemoresistance to cytarabine *via* decreased drug activation through mutation and/or downregulation of deoxycytidine kinase^[66]^. Many anticancer drugs have to be metabolically activated to produce effective cytotoxic species that will ultimately lead to the killing of cancer cells because of their cytotoxic effects. The phase Ⅰ enzymes human cytochrome P450s (CYPs) catalyze the oxidation of xenobiotics and several anticancer drugs^[67]^.

CYPs activate various chemotherapeutic agents such as procarbazine, dacarbazine, tamoxifen, ifosfamide, cyclophosphamide, thiotepa, and tegafur to produce active species that destroy cancer cells^[68]^. For example, ifosfamide and cyclophosphamide are activated by CYP2C19 and CYP2B6; the conversion of dacarbazine to active N-demethylated species is carried out by CYP1A1, CYP1A2, and CYP2E1; CYP2D6 is the enzyme that converts tamoxifen to its active form 4-hydroxytamoxifen, with assistance from CYP2B6, CYP2C9, and CYP3A4; thiotepa is converted to alkylating TEPA by CYP2B6 and CYP3A4; CYP1A2, CYP2A6, and CYP2C8 are involved in the conversion of tegafur to 5-fluorouracil^[69]^. Furthermore, CYPs have also been shown to detoxify anticancer drugs. For instance, tamoxifen, taxanes, teniposide, gefitinib, imatinib, vinca alkaloids, and sorafenib are some drugs metabolized by CYP3A4^[70]^. Another example is CYP2B6 mutations that are linked to a poor response to cyclophosphamide-based treatment in breast cancer patients, whereas CYP2D6 mutations frequently limit the anticancer effect of tamoxifen^[71]^. UDP-glucuronosyl transferases (UGTs) are enzymes involved in mediating glucuronidation reactions^[72]^. UGTs link glucuronic acid to lipophilic molecules/drugs, impairing the function of the molecules/drugs, as well as enhancing their water solubility and aiding their efflux from the cells^[73]^. One of the earliest pieces of evidence of glucuronidation's role in cancer drug resistance was reported by Gessner *et al* in 1990 when they found a relationship between high anthracycline efflux as a glucuronide conjugate from a leukemic cell line and cells resistant to daunorubicin cytotoxicity. These findings, along with two other early cell-based studies on the active metabolite of irinotecan, SN-38, and mycophenolic acid, suggested that intrinsic UGT expression and activity might have a major effect on drug sensitivity and efficacy^[74–76]^.

Recently, two novel anti-neoplastic drugs, luminespib and ganetespib, have been developed to target the heat-shock protein HSP90, which is involved in protein folding^[77]^. These inhibitors compete with HSP90 to bind to ATP and result in the proteasomal degradation of unfolded proteins. Interestingly, in a gene expression analysis study, elevated levels of UGT1A were shown to be one of the most significant differences observed between drug-resistant and drug-sensitive bladder and colorectal cancer cell lines exposed to these HSP90 inhibitors. When UGT1A was knocked down in these resistant cell lines, they became sensitive to the same inhibitors, underscoring the role of UGT1A in drug resistance^[77]^. Epigenetic mechanisms increasing the expression of UGT1A1 have also been shown, but further research is warranted^[78]^.

### Epigenetics

The term "epigenetics" can be described as changes that occur "in addition to the genetic sequence"^[79]^. The term has come to refer to any process that affects gene function without affecting the DNA sequence, resulting in changes that can be passed on to daughter cells^[80]^. Some epigenetic mechanisms have been identified, including acetylation, phosphorylation, methylation, sumoylation, ubiquitylation, chromatin remodeling, and non-coding RNA-related changes^[81]^. During the past two decades, an increasing amount of research has highlighted the importance of epigenetic mechanisms in cancer drug resistance. Hypermethylation of tumor suppressor gene sequences, leading to their silencing, or hypomethylation of oncogene DNA sequences, resulting in increased expression, is involved in the development of drug resistance^[82]^.

Histone-modifying enzymes play a crucial role in the repression or activation of gene expression by regulating chromatin architecture^[83]^. The activities of these enzymes can be changed by drug exposure, resulting in a transcriptional profile that promotes cell survival. In a recent report, SETD5 was discovered as a chromatin-based master regulator of MEK inhibitor resistance in pancreatic cancer. It has been demonstrated that once the pancreatic cells and patient-derived xenografts (PDXs) develop resistance to MEK inhibitors, they overexpress SETD5^[84]^. On the other hand, CRISPR/Cas9-based inactivation of the *SETD5* gene leads to the restoration of drug response; SETD5 lacks intrinsic histone lysine methyltransferase function, but it forms a co-repressor complex with methyltransferase G9a and histone deacetylase 3 (HDAC3) that coordinates methylation and deacetylation of histone H3 lysine 9 (H3K9)^[85]^. This complex mediates a unique gene expression profile, where genes involved in glutathione metabolism and cytochrome p450 pathway are downregulated. Furthermore, this unique expression profile promotes resistance against MEK inhibitors^[85]^. Indeed, combination therapy involving MEK inhibitors with HDAC3 and G9a inhibitors substantially reduced the growth of pancreatic PDXs while having few side effects, indicating that a similar strategy might be utilized in the clinic to extend therapeutic response in patients^[84–85]^.

Chen *et al*^[86]^ found that microRNAs and long non-coding RNAs (lncRNAs) were differentially expressed between MCF7 (a breast cancer cell line) and MCF-7/ADR (adriamycin-resistant) cells. Elevated levels of ABCB1 and reduced levels of growth arrest-specific 5 (GAS5), a lncRNA, were observed in resistant breast cancer cells and tissues. *In vitro* overexpression of GAS5 increased ADR sensitivity and apoptosis, while inhibiting efflux function and ABCB1 expression, whereas knockdown of GAS5 had the reverse effect^[86]^. Furthermore, GAS5 serves as an endogenous "sponge" that competes with miR-221-3p for dickkopf 2 (DKK2) binding, thereby inhibiting the activation of the Wingless-related integration site (Wnt)/β-catenin pathway. In addition, GAS5 improved the anti-neoplastic effect of ADR *in vivo* in terms of function. Overall, the authors showed that GAS5 played a regulatory role in ADR resistance, presumably *via* the miR-221-3p/DKK2 axis^[86]^. This approach could be further explored as a treatment strategy for breast cancer patients with chemoresistance.

Another lncRNA involved in drug resistance is urothelial cancer-associated 1 (*UCA1*)^[87]^. When cisplatin-resistant bladder cancer cells were compared with sensitive cells, the lncRNA-*UCA1* was shown to be upregulated. Upregulation of UCA1 expression boosted wingless-type MMTV integration site family member 6 (*WNT6*) mRNA and protein levels, increasing Wnt signaling and thereby cell survival^[88]^. The development of chemoresistance in high-grade serous ovarian cancer (HGSOC) has been linked to altered patterns of methylation of tumor suppressor genes. Bateman *et al*^[89]^ demonstrated that in paclitaxel-resistant HGSOC cell lines, the transcript and protein expression of AKAP12 was increased, which was linked to a reduction in levels of *AKAP12* gene methylation. *AKAP12* mRNA expression was shown to increase in cells following induced EMT, providing another putative mechanism of the chemoresistance of AKAP12 hypomethylation.

Horiuchi *et al*^[90]^ conducted a study in 2012 and demonstrated that a higher expression of S100 calcium-binding protein A4 (S100A4) was directly linked to the invasiveness of ovarian cancer cells *in vitro* as well as *in vivo*. Moreover, elevated levels of S100A4 were linked to hypomethylation of CpG sites in the first intron of S100A4 in cisplatin-resistant ovarian carcinoma cells.

Ivanova *et al*^[91]^ subjected 20 different gastric cancer cell lines to gene expression profiling, DNA methylation profiling, and drug-response tests to identify genes that govern cisplatin resistance^[91]^. It was found that *BMP4* (bone morphogenetic protein 4), an epigenetically regulated gene, was substantially expressed in cisplatin-resistant cell lines; also, *BMP4* promoter methylation levels were shown to be inversely linked to BMP4 expression in primary tumors, and patients with high BMP4-expressing tumors had a much worse prognosis; therapeutically, genetic suppression of *BMP4* resulted in considerable cisplatin sensitivity of GC cells.

The importance of epigenetic modulators in cancer treatment resistance is increasingly acknowledged, and targeting these modulators is one strategy to overcome cancer drug resistance.

### Epithelial-to-mesenchymal transition (EMT)

**Figure 3:**
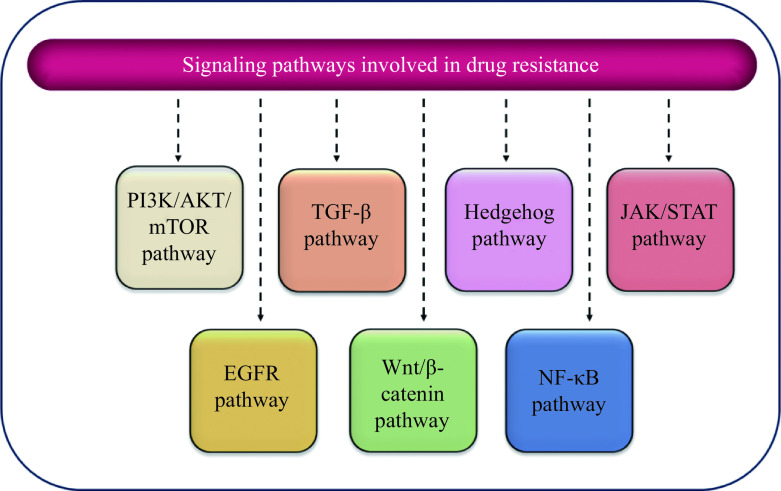
A graphical illustration of signaling pathways involved in drug resistance in cancer cells.

EMT is a fundamental reversible biological program where adherent stationary epithelial cells acquire the capability to migrate and acquire phenotypic characteristics of mesenchymal cells^[92]^. The EMT transitions occur during a variety of biological activities, which can principally be divided into three groups: (a) embryo development, (b) adult tissue regeneration, and (c) carcinogenesis^[93]^. One of the key distinctions between EMT during developmental processes and EMT during carcinogenesis is that the former is a highly orchestrated and tightly controlled process, while the latter is largely a deregulated process^[94]^. One of the primary consequences of EMT is the ability of solid tumors to spread from one place to another known as metastasis^[95]^. Metastasis is a highly sophisticated process that includes alterations in both cancer cells and surrounding stromal cells. Furthermore, the formation of new blood vessels known as angiogenesis also occurs around metastatic tumors. Upon activation of the EMT program in cancer cells, multiple changes occur which include: (a) disruption of cell-cell junctions, (b) changes in cell polarity occurring from apicobasal to front-rear polarity, (c) degradation of the underlying basement membrane, (d) reorganization of the extracellular matrix, (e) reorganization of actin stress fibers, (f) loss of expression of cytokeratins and E-cadherin, and (g) upregulation in the expression of mesenchymal markers such as vimentin, fibronectin, N-cadherin, β1, and β3 integrins. The transcription factors that play key roles in the EMT process are the basic helix-loop-helix factors TWIST1 and TWIST2, zinc-finger E-box binding homeobox factors ZEB1 and ZEB2, as well as SNAIL (SNAI1) and SLUG (SNAI2)^[96]^. The role of EMT in cancer drug resistance was established approximately three decades ago, and its importance in a variety of malignancies, including pancreatic, lung, breast, and bladder cancer, has since been recognized. Signal transduction pathways such as Hedgehog (Hh), TGF-β, NOTCH, and Wnt have been demonstrated to activate EMT; however, they are also known to induce cancer drug resistance^[97]^. ***Fig. 3*** depicts some of the most common pathways involved in drug resistance.

Among the various drugs used to treat metastatic breast cancer, taxanes are commonly employed^[98]^. Docetaxel and paclitaxel are the two most frequently used taxanes^[99]^. Both of these drugs disrupt microtubules and induce mitochondrial-mediated apoptosis^[100]^. However, resistance to these drugs has been reported in most breast cancer patients and well-established breast cancer cell lines^[101]^. The acquired resistance has been shown to be associated with the EMT phenotype. The connection to the EMT phenotype was demonstrated by comparing biopsies obtained before and after taxane (docetaxel or paclitaxel) and anthracycline treatment regimens, where a clear upregulation of breast cancer stem cell (BCSC) genes and TGF-β was noticed in chemotherapy-treated tumors^[101–102]^. Treatment with LY2157299 (a TGF-β type I receptor inhibitor) led to the suppression of paclitaxel's ability to induce the expression of IL8 and its activation of BCSCs' self-renewal and expansion. In mice with SUM159 tumors, this treatment plan prevented disease recurrence^[102]^. These data indicate that inactivating TGF-β-driven EMT processes might provide novel avenues for treating taxane resistance in clinical settings.

The role of EMT in the upregulation of TWIST1 and SNAIL has been shown to provide resistance against doxorubicin by inducing P-gp^[103]^. Similarly, upregulation of PARP1 by SNAIL in MDA-MB-231 breast cancer cell lines contributes to resistance against doxorubicin. Treatment with ABT-888 (a PARP inhibitor) led to an increase in apoptosis of cells. These results indicate that targeting SNAIL expression may be one of the ways to inhibit EMT-based drug resistance^[104]^. The crucial role imparted by EMT mechanisms in causing diverse cancers is now well established. Therefore, targeting the components of EMT would be one of the ways to tackle cancers, and further research in this field is necessary.

### Deregulation of cellular energetics

Because of their continuous proliferation and high growth rate, cancer cells need a constant source of energy^[105]^. This increased energy demand is supplemented by rapid adaptation to different metabolic pathways. Cancer cells particularly depend on glycolysis for energy production, but they also exhibit increased glutamine metabolism rates and enhanced fatty acid synthesis. Recent studies suggest that these characteristics of cancer cells including Warburg-like glucose metabolism, glutaminolysis, and fatty acid production are associated with drug resistance^[106–109]^.

For example, the human gastric cancer cell lines SNU-620 and SNU-638 were used by Yoo *et al* to establish 5-fluorouracil (5-FU) and cisplatin-resistant cell lines^[110]^. Protein extracts from the parental as well as drug-resistant derivative lines were used to test for proteins linked to drug resistance using two-dimensional gel electrophoresis (2-DE) and matrix-associated laser desorption ionization-mass spectrometry (MALDI-MS). Pyruvate kinase M2 (PK-M2) was identified as one of the proteins showing decreased expression in cisplatin-resistant cell lines in comparison to the parental cells. Antisense oligonucleotide-based repression of PK-M2 led to acquired resistance against cisplatin in SNU-638 cells. Furthermore, 11 different gastric cancer cell lines tested for PK-M2 activity showed a positive correlation with cisplatin sensitivity. In conclusion, these data indicate that PK-M2 is associated with cisplatin chemoresistance^[110]^.

In another example, it was shown that tamoxifen and lapatinib by promoting glycolysis induce resistance in breast cancer cells^[111–113]^. Liu *et al*^[114]^ demonstrated that elevated levels of hexokinase 2 induced tamoxifen resistance in MCF-7 cells. One of the key enzymes in glycolysis is lactate dehydrogenase A (LDHA) which converts pyruvate to lactate and has been reported to be involved in paclitaxel resistance in breast cancer^[109]^. In colon cancer^[115]^ and cervical cancer, pyruvate dehydrogenase kinase 3 contributes to hypoxia-induced treatment resistance^[116]^. In gastric cancer, mTORC1 signaling is activated by glutaminolysis and is associated with cisplatin resistance^[116]^. Fatty acid synthase (FASN), a crucial enzyme involved in the synthesis of fatty acids, is associated with trastuzumab resistance in breast cancer. Furthermore, radioresistance and resistance against gemcitabine in pancreatic cancer have also been reported^[116]^.

Therefore, depriving cancer cells of glucose has catastrophic consequences on their survival^[117–118]^. This can be achieved by targeting the enzymes and transporters involved in the glucose pathway. In some cases, targeting the glucose pathway also helps in overcoming drug resistance, as depicted by the inhibitors developed to target various enzymes in the glucose pathway.

### Immune evasion/avoiding immune destruction

**Figure 4:**
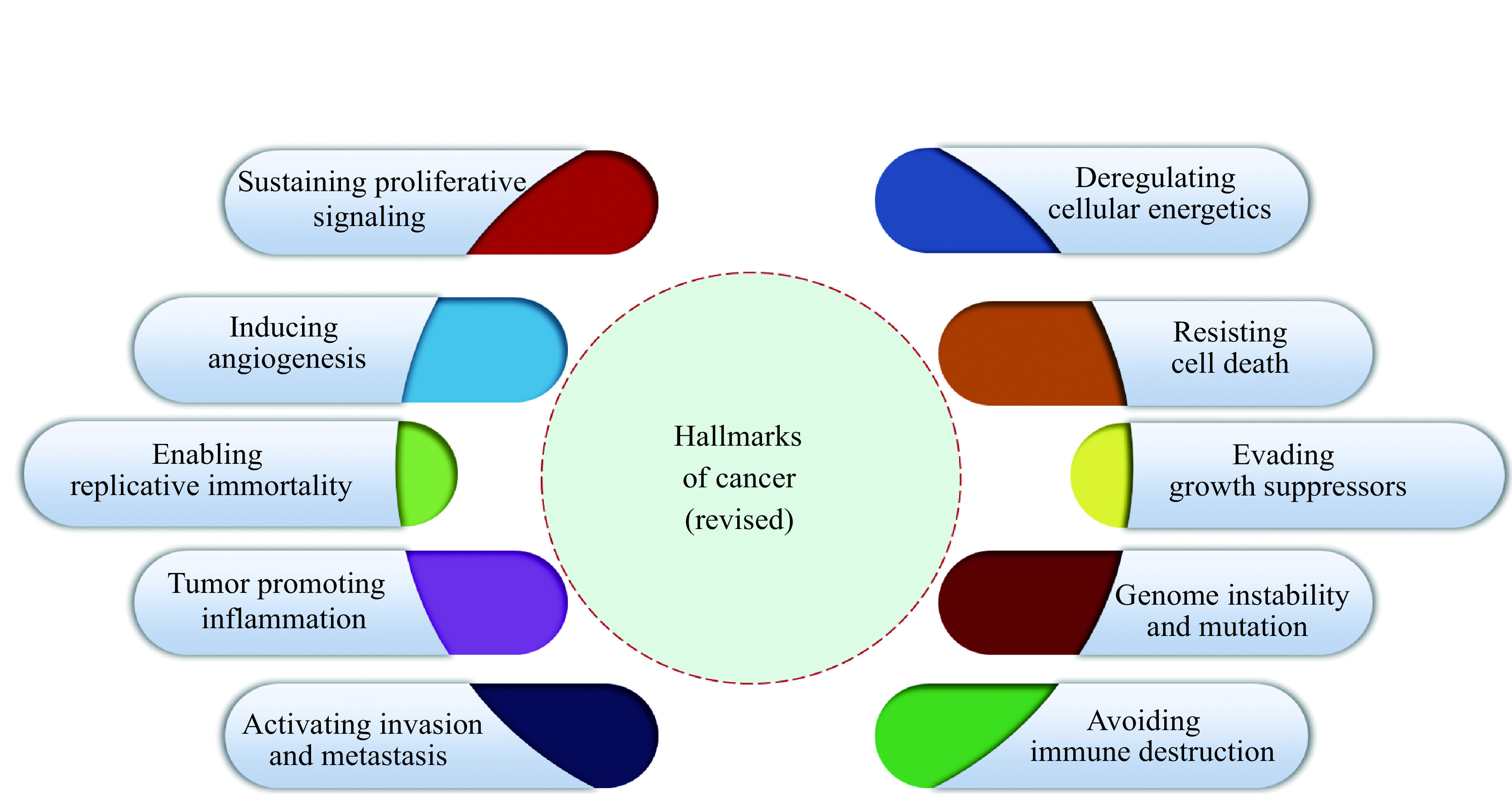
Graphical illustration of "revised" hallmarks of cancer.

The crucial role imparted by the immune system in the recognition of harmful agents, foreign bodies, viruses, bacteria, abnormal cells, and cancer cells, as well as the orchestrated elimination of cancer cells, is well established^[119]^. Despite this tight surveillance, some cancer cells can evade immune recognition and continue to proliferate and spread^[5]^. Among the various drug resistance modes exhibited by cancer cells, the immune evasion depicted in ***Fig. 4*** is one of the highly intricate processes *via* which cancer cells develop ways to avoid detection and destruction by the immune system, making some cancer treatments less effective. Multiple mechanisms have been demonstrated by which cancer cells avoid immune destruction including: (a) downregulation of antigen presentation^[120]^, (b) loss of tumor antigens^[121]^, (c) immune checkpoint activation^[122]^, (d) tumor microenvironment^[122]^, (e) epigenetic changes^[123]^, (f) tumor heterogeneity^[124]^, (g) influence of microbiome^[125]^, (h) immune escape variants; (i) immunosuppressive pathways^[126]^, and (j) treatment-induced changes^ [122]^. However, discussing the details of all these mechanisms is beyond the scope of this review, and readers are directed to excellent reviews by Kim and Cho^[122]^ and Dutta *et al*^[127]^.

One study by Bai *et al*^[128]^ has shown that adoptive therapy with both monoclonal and polyclonal transgenic cytotoxic T lymphocytes that are specific for the P1A tumor antigen selects for a number of mutations in the P1A antigenic epitope; consequently, by altering the interaction between major histocompatibility complex (MHC) and peptides, as well as T-cell receptor binding to the MHC:peptide complex, these alterations significantly reduced T-cell recognition of the tumor antigen; as a result, antigenic drift is one of the mechanisms through which tumor cells evade immune surveillance.

Indoleamine 2,3-dioxygenase (IDO) synthesis within the tumor microenvironment in response to IFN-γ synthesized by anti-tumor T cells is one example of treatment-induced immune resistance observed in cancers^[129]^. The enzyme IDO promotes T-cell suppression by catalyzing the conversion of tryptophan to kynurenine. Both preclinical data^[130]^ and an ongoing clinical study (NCT01604889) examining a combination therapy with the anti-cytotoxic T-lymphocyte-associated protein 4 (CTLA-4) antibody ipilimumab and INCB024360, a selective inhibitor of IDO, are based on the idea that IDO plays a role as an immune resistance mechanism to immune checkpoint therapy, including anti-CTLA-4 and anti-PD-1 blocking antibodies^[131]^.

One study by Spranger *et al*^[132]^ reported that tumor-intrinsic β-catenin activation by downregulation of CCL4 expression might inhibit the recruitment of CD103^+^ dendritic cells and infiltration of T cells into the tumor microenvironment. The activation and cytotoxic effects of effector T cells are reduced because no antigen can be presented to T cells and DCs are blocked from reaching the tumor microenvironment^[132]^.

Peng *et al*^[133]^ demonstrated in preclinical models of melanoma that loss of PTEN in tumor cells reduced T cell trafficking into tumors and impaired T cell-mediated tumor killing; furthermore, PTEN deficiency in patients was linked to a reduction in T-cell infiltration at tumor sites and a worse PD-1 inhibitor response; additionally, loss of PTEN in tumor cells led to an enhanced production of immune-suppressive cytokines and inhibited autophagy, which in turn led to decreased T cell-mediated cell deaths; notably, the effectiveness of anti-CTLA-4 as well as anti-PD-1 antibodies in mouse models could be enhanced *via* treatment with a selective PI3Kβ inhibitor. The authors conclude that loss of PTEN leads to immune resistance and provides evidence to explore combination therapies involving immunotherapies and PI3K-AKT pathway inhibitors.

Taken together, cancer immune evasion is a highly intricate process, and addressing it through monotherapy or a combination of therapies, including immunotherapy, would be helpful in tackling drug resistance.

### Tumor-promoting inflammation

Inflammation is a complex defense mechanism that involves the activation, recruitment, and orchestrated elimination of pathogenic and harmful entities from the body^[134]^. Research in the last decade has established a link between inflammation and its functions in tumor initiation, progression, and metastasis in diverse cancers. Inflammation has also been shown to play a pivotal role in tissue repair, remodeling, and regeneration^[135]^. In cancers, inflammation regulates processes such as DNA damage response, autophagy, apoptosis, and the tumor microenvironment including cancer-associated fibroblasts (CAFs) and tumor-associated macrophages (TAMs). Inflammation brings about its effect through recruiting various immune cells that produce a cocktail of pro- (TGF-β, IL-4, IL-10, and IL-13) and anti-inflammatory (IL-1β, IL-2, IL-6, IL-8, IL-17, TNF-α, and IFN-γ) cytokines. Interactions between pro- and anti-inflammatory cytokines create a complex network whose dynamic equilibrium controls how inflammation develops and manifests. Inflammation has been found to modulate critical signaling pathways involved in tumorigenesis such as Wnt, Toll-like receptor, Janus kinase (JAK)/signal transducer and activator of transcription 3 (STAT3), and nuclear factor kappa-light-chain-enhancer of activated B cells (NF-κB) signaling^[136]^. The role of inflammation in drug resistance is also being delineated, for example, inflammation regulates the expression of several drug efflux transporters such as ABCB1, ABCC1, and ABCG2, as well as drug metabolism enzymes like CYP1A2 and CYP3A4^[137]^.

Qiao *et al*^[138]^ found that CAF-derived interleukin 6 (IL-6) conferred cisplatin resistance in esophageal squamous cell carcinoma (ESCC) by upregulating C-X-C motif chemokine receptor 7 (CXCR7) expression *via* STAT3/NF-κB pathways. Knockdown of CXCR7 led to a reduction in proliferation and chemoresistance induced by IL-6; furthermore, silencing CXCR7 led to a drastic reduction in gene expression profile related to stemness, chemoresistance, and EMT, as well as suppressing ESCC cell proliferation in angiogenesis assay and three-dimensional culture systems^[138]^. These findings indicate that the IL-6-CXCR7 axis could be therapeutically exploited for treating ESCC^[138]^.

Several recent studies showed that IL-6 *via* activation of STAT3^[139–140]^ induced resistance against tyrosine kinase inhibitors (TKIs) in NSCLC^[141]^. In one study, Yao *et al*^[142]^ demonstrated that in H1650 cells (drug-resistant bronchoalveolar metastatic carcinoma), TGF-β was primarily responsible for resistance against erlotinib and enhanced IL-6 axis activation. In another study, Zhong *et al*^[143]^ used MEDI5117, an IL-6 targeting antibody, and showed that it might enhance the efficacy of gefitinib in trastuzumab-resistant tumors. Similarly, one study by Wang *et al*^[144]^ demonstrated that A549/DDP cells (cisplatin-resistant) expressed high levels of drug-resistant proteins like P-gp and acquired an EMT phenotype; furthermore, shRNA-based knockdown and the use of TGF-β inhibitors led to an increase in sensitivity to cisplatin.

The NF-κB pathway is activated in CAFs in pancreatic ductal adenocarcinoma (PDAC) by robustly expressing IL-1 receptor-associated kinase 4 (IRAK4), thereby leading to survival, proliferation, and chemoresistance of PDAC cells; cytokine profiling of CAFs led to the identification of IL-1β as one of the main cytokines that activated IRAK4 in CAFs; furthermore, inhibition of IRAK4 and IL-1β led to PDAC tumors being less fibrotic and sensitive to gemcitabine^[145]^. One of the standard drugs for the treatment of hepatocellular carcinoma is sorafenib. However, because of the development of resistance, the drug is no longer effective in patients. Kang *et al*^[146]^ knocked down the expression of TGF-β through shRNA-based technology in these cells, making them sensitive to sorafenib treatment again.

Collectively, all these findings point out the significant role played by inflammation in drug resistance. Consequently, targeting inflammation should be considered for cancer treatment.

### Genome instability and mutations

For any cell, the maintenance of cellular integrity is achieved through the surveillance of genomic stability. The more stable the genome, the higher the cellular integrity. On the other hand, cancer cells often lose their cellular integrity because of unstable genomes; furthermore, because of this genomic instability, cancer cells enjoy a wide range of advantages such as having shorter cell cycles and bypassing immune and intracellular regulatory mechanisms, giving cancer cells a growth advantage over normal cells^[147]^. Genomic instability encompasses base pair variations, microsatellite instability, or changes in the number of chromosomes or their structure also known as chromosome instability (CIN)^[148]^. Genomic instability has been linked to poor prognosis in cancer patients and drug resistance^[149]^.

Replogle *et al*^[150]^, for instance, showed that aneuploidy delayed cell cycle progression, notably from the G1 to the S phase, and induced cellular stress; additionally, a single chromosome gain could lead to drug resistance against paclitaxel and cisplatin by reducing their ability to damage DNA and microtubules; moreover, G1 cell cycle delays were enough to make euploid cells more resistant to chemotherapy. Finally, the correlation between aneuploidy with delayed proliferation and drug resistance was validated using datasets available in the Cancer Cell Line Encyclopedia^[150]^. In conclusion, the findings showed that aneuploidy decreased cell proliferation, which resulted in a selective advantage for cancer cells during chemotherapy^[150]^.

In a recent study, Hanjani *et al*^[151]^ performed multi-region whole-exome sequencing on 100 early-stage NSCLC tumors that were surgically removed from patients before receiving any systemic treatment. To understand and establish evolutionary histories, gather a census of sub-clonal and clonal occurrences, and evaluate the link between intratumor heterogeneity and recurrence-free survival, they sequenced and analyzed 327 tumor areas; and found high intratumor heterogeneity for both mutations as well as alterations in copy number; furthermore, such intratumor heterogeneity was found to be linked to genome doubling and continued dynamic CIN, resulting in the simultaneous development of driver somatic copy-number changes, including amplifications in *BCL11A*, *CDK4*, and *FOXA1*; in conclusion, the authors reported that chromosomal instability might be used as a prognostic tool in NSCLC, and intratumor heterogeneity mediated *via* CIN was linked to enhanced risk of recurrence and death^[151]^.

Penner-Goeke *et al*^[152]^ evaluated CIN in two ovarian cancer cell models of drug resistance (PEO1/4 and A2780s/cp) as well as in serial samples taken from the ascites of five patients with epithelial ovarian cancer (EOC); they primarily focused on resistance and recurrent illness by quantifying and comparing the chromosomal instability score (CS) across the patient samples to understand the dynamics of CIN within EOC. By employing single-cell studies, a significant difference in nuclear size and CS value was observed in all the EOC patient samples; in conclusion, their data indicate that CIN can be used as a biomarker for EOC progression that is linked to drug resistance^[152]^.

Finally, it is necessary to conduct further research to understand the function of genomic instability and how to target it to combat drug resistance.

## Functional role of microRNAs in cancer drug resistance

**Figure 5:**
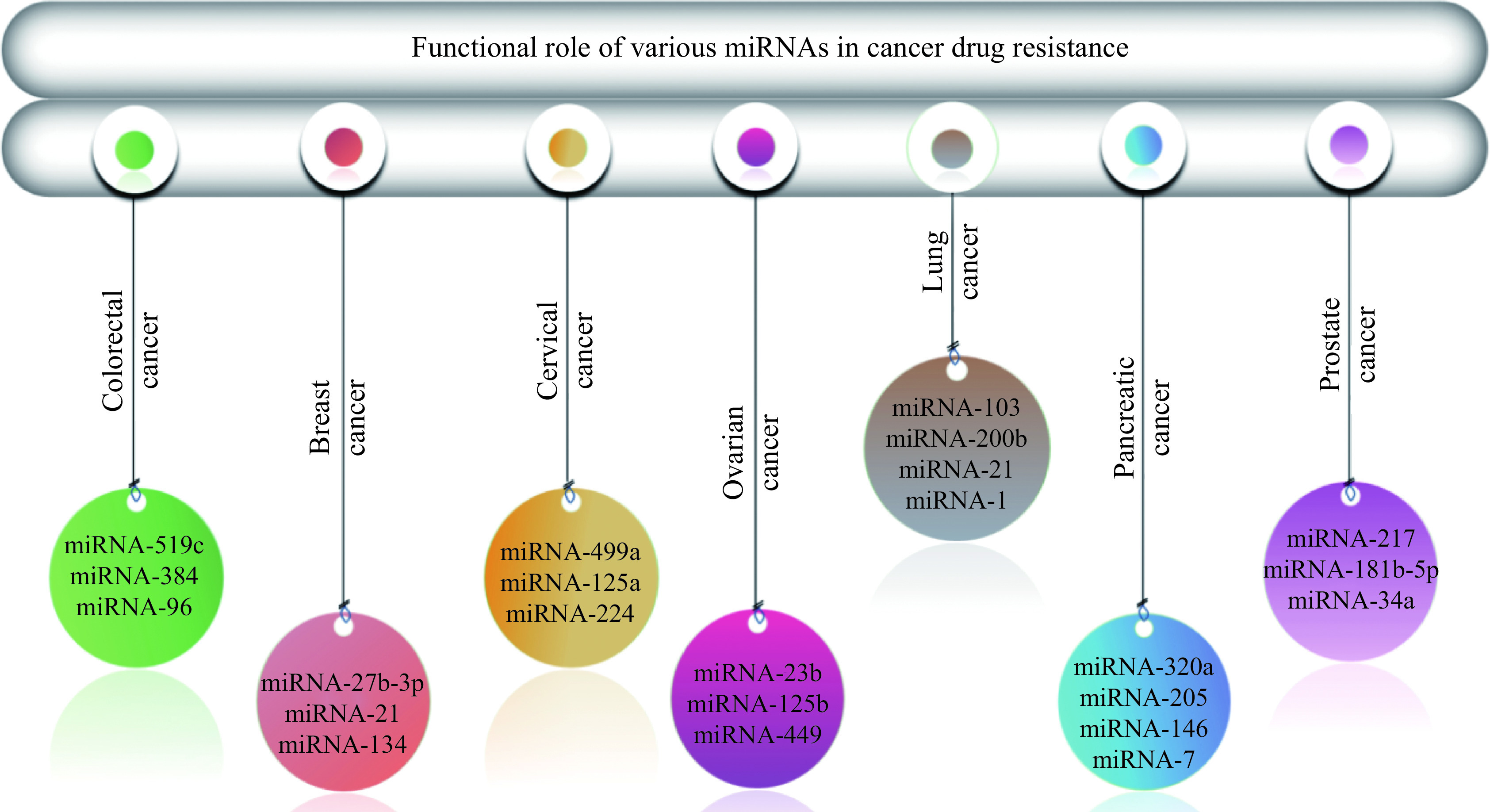
Graphical representation of microRNAs in cancer drug resistance.

MicroRNAs (miRNAs) can be described as nonprotein-coding RNAs^[153–154]^. They range from 18 to 24 nucleotides in size and play important roles in several biological processes, including the cell cycle, cell proliferation, cell death^[155]^, fat metabolism^[156]^, and immune response^[157]^. miRNAs can serve as oncogenes or tumor suppressors influencing tumor development and invasion^[158]^. In addition, miRNAs are involved in the modulation of multiple protein-coding genes in cancer and cancer drug resistance, as depicted in ***Fig. 5*** and ***Table 3***.

**Table 3:** Chemoresistance induced by miRNAs in myriad cancers and their specific targets

Chemotherapy agents	Targets	Tumor/cancer	miRNAs	References
Adriamycin	MDR1/MRP1	Glioma	miR-127	[193]
Anthracyclines	MDR1	SCLC	miR-7	[194]
Bortezomib	BAFF	Multiple myeloma	miR-202	[195]
Cisplatin	PTEN, MDR1/MRP1, Cyclin D1, GRB2, ERK2, RSK1, and RSK2	NSCLC Ovary	miR-181a miR-196a miR-634	[196]
Dexamethasone	BAFF	Multiple myeloma	miR-202	[195]
Doxorubicin	P-gp and MRP1/ABCC1	Gastric Breast	miR-103 miR-107 miR-134	[196]
EGFR inhibitors	KRAS and AKT1	NSCLC	miR-4689	[197]
Epirubicin	ABCG2	Breast	miR-25	[198]
Melphalan	MRP1/ABCC1	Multiple myeloma	miR-221 miR-222	[199]
Paclitaxel	PTEN and p-gp/ABCB1	Ovary NSCLC	miR-17-5p miR-145 miR-181a	[196]
Thalidomide	BAFF	Multiple myeloma	miR-202	[195]
Trastuzumab	PTEN and PDCD4	Breast	miR-21	[200]

**Table 3:** Chemoresistance induced by miRNAs in myriad cancers and their specific targets (continued)

Chemotherapy agents	Targets	Tumor/cancer	miRNAs	References
Temozolomide	MDR1/ABCG2	Glioblastoma	miR-9	[201]
Vincristine/cisplatin	ABCB1	Gastric	miR-129-5p	[202]
Vincristine/oxaliplatin/cisplatin	P-gp/ABCB1	Colorectal Gastric	miR-200c miR-508-5p	[196]
5-Fluorouracil mitomycin C	P-gp/ABCB1	Colorectal	miR-200c	[203]
5-Fluorouracil	ABCB1, P-gp/ABCB1, and ABCG2	Gastric Colorectal	miR-129-5p miR-508-5p miR-519c	[196]
Abbreviations: ABCB1, ATP-binding cassette sub-family B member 1; ABCC1, ATP binding cassette subfamily C member 1; ABCG2, ATP binding cassette subfamily G member 2; AKT1, serine/threonine kinase 1; BAFF, B-cell activating factor belonging to the TNF family; EGFR, epidermal growth factor receptor; ERK2, extracellular signal-regulated kinase; GRB2, growth factor receptor bound protein 2; miR, microRNA; MRP1, multidrug resistance protein 1; MDR1, multidrug resistance 1; NSCLC, non-small cell lung cancer; KRAS, Kirsten rat sarcoma virus; PDCD4, programmed cell death 4; P-gp, permeability glycoprotein; PTEN, phosphate and tensin homolog; RSK1: ribosomal protein S6 kinase A1; SCLC, small cell lung cancer.				

The principal mode of action of miRNAs is that they bind to the seed region at the 3′-end or the 5′-end of the target mRNA and lead to gene silencing or degradation of the target mRNA. However, the interaction of miRNAs with promoter sequences of the target gene has also been reported, leading to the induction of transcription^[159]^.

One of the extensively studied and well-understood miRNAs contributing to chemoresistance in breast cancer is miRNA-21, and overexpression of miR-21, which functions as an oncogene, inhibits tumor suppressor genes and has been linked to a poor prognosis in breast cancer patients. For example, a study by Wang *et al*^[160]^ reported that high expression levels of miR-21 and downregulation of the tumor suppressor gene (*PTEN*) were found in breast cancer cells resistant to doxorubicin (MCF-7/ADR) compared with the parental MCF-7 cells. Inhibiting miR-21 by a mimic or inhibitor leads to the sensitivity of resistant cells (MCF-7/ADR) to doxorubicin; furthermore, an increased caspase-3 activity was observed, which was linked to an enhanced apoptosis of MCF-7/ADR cells upon the use of inhibitors for miR-21^[160]^.

In a study conducted in 2018, Chen *et al*^[161]^ reported a decrease in the expression levels of miRNA 27-b-3p in breast cancer cell lines and tissues, and further mechanistic analysis showed that miRNA-27b boosted PTX (paclitaxel) responses by targeting *GRB2* and *CBLB*, inactivating both the MAPK/ERK and PI3K/AKT signaling pathways. These findings suggest that miR-27b is disadvantageous for tumors and chemotherapy-induced resistance, indicating that miRNA-27b can be used as a prognostic molecular marker in breast cancer patients.

Lu *et al*^[162]^ in 2015, showed that miR-134 was differentially expressed between doxorubicin-sensitive and doxorubicin-resistant breast cancer tissues and that miR-134 expression was reduced in doxorubicin-sensitive breast cancer in comparison to that of doxorubicin-resistant breast cancer. Furthermore, they developed a doxorubicin-resistant MCF-7/ADR (adriamycin) breast cancer cell line and found that overexpression of miR-134 significantly reduced cell proliferation and increased cell apoptosis in this cell line^[162]^. These findings demonstrate that doxorubicin-resistant breast cancers are associated with a higher level of miR-134 suppression. ABCC1 (MRP1) is a substrate for doxorubicin, and previous reports have demonstrated that ABCC1 is linked to doxorubicin resistance in multidrug-resistant cancer cells^[163]^. ABCC1 expression was elevated to a larger extent in doxorubicin-resistant breast cancer samples than in doxorubicin-sensitive breast cancer tissues, and it was highly upregulated in MCF-7/ADR cells. Overexpression of miR-134 resulted in a significant reduction in the ABCC1 expression. All these findings suggest that miR-134 downregulation promotes doxorubicin resistance in breast cancer and that the process is mediated through the regulation of the *ABCC1* gene, which should be further explored in a therapeutic setting^[162]^.

Ge *et al*^[164]^ demonstrated the role of miR-96 in oxaliplatin-resistant colorectal cancer (CRC) cells. By using gene expression studies, the authors identified tropomyosin 1 (*TPM1*) as a direct target of miR-96; furthermore, the authors reported an upregulation of miR-96 but a downregulation of *TPM1* in CRC, compared with normal adjacent tissues^[164]^. The luciferase reporter assay showed a reduction following transfection with miR-96 mimics and luciferase reporter plasmid containing the wild-type sequence of the 3′-untranslated region of *TPM1*; additionally, knockdown of miR-96 enhanced the chemosensitivity of CRC cells to oxaliplatin by targeting *TPM1*; in conclusion, miR-96 modulates oxaliplatin resistance in CRC cells^[164]^.

Park *et al*^[165]^ performed miRNA profiling and identified miR-96-5p upregulation in tumors from patients with sunitinib-resistant renal clear cell carcinoma; further analysis led to the identification of PTEN as a target of miR-96-5p; in The Cancer Genome Atlas renal clear cell carcinoma cohort, a negative correlation between miR-96-5p and PTEN was found; furthermore, higher expression of miR-96 and lower levels of PTEN were linked to a poor prognosis; in the 3′-untranslated region (3′ UTR) region of *PTEN*, a novel miR-96-5p binding site and a direct suppression were identified through the luciferase reporter assays; additionally, the miR-96-5p-based PTEN repression demonstrated an increased migration and proliferation in sunitinib-treated cell lines.

Mencia *et al*^[166]^ used microarray studies to identify differentially expressed miRNAs in MTX-sensitive and MTX-resistant HT29 colon cancer cells. The authors reported that miR-224 was identified as one of the highly differentially expressed miRNAs based on raw signal values; by using bioinformatics analysis, putative targets were identified including CDS2, DCP2, HSPC159, MYST3, and SLC4A4; furthermore, an anti-miR against miR-224 was used to desensitize the cells to MTX, simulating the resistant phenotype; additionally, siRNAs against SLC4A4 or PPRH hairpin incubation against CDS2 or HSPC159 increased sensitivity to MTX^[166]^. These outcomes demonstrate that miR-224 and its targets contribute to MTX resistance in HT29 colon cancer cells.

Zhou *et al*^[167]^ investigated the role of miR-449a in cisplatin-resistant ovarian cell lines SKOV3/DDP and A2780/DDP, and compared it with their sensitive parent lines SKOV3 and A2780. The authors found that overexpression of miR-449a led to enhanced cisplatin sensitivity of SKOV3/DDP and A2780/DDP by inhibiting proliferation and promoting apoptosis; furthermore, luciferase assays confirmed that miR-449a functioned by repressing NOTCH1; in line with this, BALB/c nude mice that received intraperitoneal injections of SKOV3/DDP cells that had been transfected with miR-449a mimics showed improved *in vivo* cisplatin sensitivity^[167]^. These findings point to a potential therapeutic approach for the treatment of cisplatin resistance in ovarian cancer that involves the ectopic expression of miR-449a.

Feng *et al*^[168]^, through their gene expression data, identified miR-200b as the most down-regulated miRNA in docetaxel-resistant SPC-A1/DTX cells, compared with their sensitive parental SPC-A1 cells. They reported that the expression of miR-200b ectopically reversed docetaxel-induced chemoresistance of lung adenocarcinoma cells; furthermore, luciferase reporter assays containing a 3′ UTR sequence of *E2F3* mRNA showed that miR-200b could directly target E2F3^[168]^. These results suggest that down-regulation of miR-200b could lead to E2F3 overexpression and in turn, contribute to the chemoresistance of lung adenocarcinoma cells to docetaxel.

In 2014, Fang *et al*^[169]^ showed that miR-200b regulated drug resistance in small-cell lung cancer (SCLC). The results showed that ectopic expression of miR-200b targeted zinc finger E-box-binding homeobox 2 (ZEB2) and rendered SCLC sensitive to chemotherapeutic drugs such as cisplatin, adriamycin, and etoposide; furthermore, the expression of miR-200b was downregulated in resistant cells in comparison to the sensitive parental cells^[169]^.

Meng *et al*^[170]^ investigated the role of miRNAs in gemcitabine (GEM) resistance in pancreatic cancer. Through microarray experiments, the authors found that miR-146a-5p expression was decreased in PDAC tissues, compared with the adjacent normal tissues; furthermore, *in silico* and *in vitro* experiments identified tumor necrosis factor receptor-associated factor 6 (TRAF6) as a direct target of miR-146a-5p; by targeting the 3′ UTR of TRAF6, it was shown that miR-146a-5p reduced PDAC cell growth and sensitized the cells to GEM treatment^[170]^. These results indicate that miR-146a-5p is involved in GEM chemoresistance in pancreatic cancer.

Pan *et al*^[171]^ studied the role of drug resistance mediated by miR-217 in TKI-resistant CML cells. It was shown that in K562 dasatinib-resistant cells (K562DR), miR-217 was found to interact with pro-oncogenic anterior gradient 2 (AGR2). Decreased miR-217 levels were shown to be associated with an increased expression of AGR2 in K562DR cells. Furthermore, a negative correlation between miR-217 expression and AGR2 expression was demonstrated by luciferase reporter assays. Expression of miR-217 forcedly led to a reduction in AGR2 expression levels and hampered the ability of K562DR cells to resist TKIs. In a mouse xenograft transplantation model, overexpression of miR-217 re-sensitized K562DR cells to dasatinib therapy. Altogether, they showed that drug resistance brought on by TKI therapy was associated with miR-217. All the above-given examples explain the functional role played by miRNAs in drug resistance in various cancers, emphasizing their significance and targeting them can be one of the useful strategies for treating cancers.

## Therapeutic strategies to overcome drug resistance

Treating drug-resistant cancers has become a herculean challenge for both scientists and clinicians in the 21st century. Although novel anticancer drugs are introduced in the market from time to time, unfortunately, after a period of initial response to these drugs, most of the cancers develop resistance. In addition, the side effects and toxicities attached to these drugs are of major concern. Therefore, the quest to find and develop novel therapeutic strategies targeting drug-resistant cancers is essential. The following paragraphs discuss some of the strategies that have been developed to overcome cancer drug resistance.

### Combination therapy

The distinguishing feature of cancer cells is their multiclonal origin and genetic heterogeneity. Therefore, it is imperative that monotherapy targeting a single pathway, although still practiced, would not be very effective. Furthermore, continuous treatment with a single agent enhances the risk of drug resistance, because cancer cells activate alternative rescue pathways for survival. Combination therapy, on the other hand, can target multiple signaling pathways simultaneously, making it more effective than monotherapy. The key benefit of combination therapy is the synergistic interaction of the drugs, which can effectively eliminate the cancer cells, while also requiring lower drug dosage, thereby reducing drug-induced side effects. For example, the combination therapy of prednisone, vincristine, and 6-mercaptopurine (POMP regimen) was shown to be effective in reducing the tumor burden of pediatric patients with ALL and prolonged remission^[172]^. In a clinical trial (NCT01281696), the combination therapy of cisplatin, etoposide, and bevacizumab showed a promising efficacy in metastatic breast cancer patients^[173]^. The BeTa (bevacizumab/tarceva) trial study^[174]^, where a combination of erlotinib and bevacizumab was used in treating advanced NSCLC, showed a doubling of progression-free survival in comparison to erlotinib alone, although it had no effects on overall survival^[174–175]^. In another clinical trial (NCT00967031), the combination of capecitabine and lapatinib was shown to be effective as a first line of therapy for HER2-positive breast cancer patients with brain metastases^[176]^.

### The "on and off" strategy

Traditionally, cancer patients were treated with the highest possible dose of the drug, also known as the maximum tolerated dose (MTD), that would not cause any severe side effects. However, it has become clear that a treatment approach like that may lead to the development of drug resistance because it constantly presses tumors to favor cancer cells with a high drug resistance. Therefore, the "on and off" strategy was developed, where patients are given the drug intermittently. Alternatively, a high dose of chemotherapeutic drug was given followed by a low dose. This kind of treatment may facilitate the competition between sensitive and resistant cells, which may prevent the formation of drug-dependent resistant cells^[177]^. This phenomenon is observed in ALK kinase inhibitor-treated lymphoma cells that developed a dependence on it, indicating that intermittent dosage may prolong control of ALK-positive tumors^[178]^. Another example of this is the BRAF and MEK-resistant melanoma cells that became sensitive upon withdrawal of the drug^[179]^.

### Chemical strategies to overcome drug resistance

Several chemical strategies have been developed to combat resistance against targeted anticancer therapies. These strategies proposed by Pisa and Kapoor^[180]^ include: (a) Designing drugs targeting multiple binding pockets; (b) Designing drugs with distinct binding modes; (c) Designing cysteine-targeting covalent inhibitors; (d) Designing inhibitors targeting allosteric binding sites; (e) Crash testing drugs; and (f) Overcoming resistance by targeted degradation. Discussion on these topics is beyond the scope of this article and for further details, please refer to the review by Pisa and Kapoor^[180]^.

### Nanotherapeutics

Nanotherapeutics and the application of nanomaterials in the field of medicine have transformed the healthcare sector. The primary concerns associated with a lot of chemotherapeutics are the development of resistance, drug-related toxicities, and the inability to achieve therapeutic efficiency. To address these concerns, nanomaterials ranging from 1 to 1000 nm have been explored and modified in such a way that they can traverse through various biological barriers, thereby enhancing drug permeability and drug retention, resulting in better treatment outcomes. Furthermore, nanotherapeutics have superior pharmacokinetic properties, bioavailability, and more biocompatibility in comparison to general chemotherapeutics. Several specialized medical fields have benefited from the use of nanotherapeutics, including infectious diseases, blood disorders, diabetes, neurodegenerative diseases, orthopedic problems, and cancer.

In certain cancers, the use of nanotherapeutics has been shown to overcome chemotherapy-induced resistance. One of the first and well-known examples of nanotherapeutics is Doxil. Doxil is a liposomal encapsulated version of doxorubicin, exhibiting lower cardiotoxicity and a better half-life than normal doxorubicin. Furthermore, Doxil has also been approved for clinical trials as an anticancer nanomedicine^[181]^. In another study, chitosan gold nanoparticles were tested on CEM (T-acute lymphocytic leukemia) and K562 (chronic myeloid leukemia) cell lines. These nanoparticles induced the production of reactive oxygen species, thereby leading to the loss of mitochondrial membrane potential and having detrimental effects on cancer cells but without any negative effect on normal immune cells^[182]^. Kuo *et al*^[183]^ developed curcumin (CCM)-loaded chitosan-poly (lactic-co-glycolic acid) (PLGA) nanoparticles modified with sialic acid to permeate the blood-brain barrier. This formulation was demonstrated to inhibit the proliferation of glioblastoma cells and brain cancer stem cells.

Some other nanotherapeutic strategies to overcome drug resistance include: (a) Development of novel pH-responsive nanoparticles based on tumor acidity; (b) Nanoparticles targeting the hypoxic tumor microenvironment; (c) Co-delivery of nano-formulations for reversal of resistance mechanisms; (d) Enhancing drug delivery by antibody-modified active targeting; (e) Combining strategies in reversal of drug resistance; and (f) Interaction of chemotherapeutics and metastasis-related inhibitors. The readers are directed to Cao *et al* for further details on these topics^[184]^.

Lately, next-generation sequencing techniques like bulk RNA-seq and single-cell RNA-seq could be very helpful in determining the driver mutations in a mixed population or single populations of cancer cells, thereby helping in targeting specific signaling pathways^[185]^. Apart from the above-discussed therapeutic strategies, the use of oncolytic viruses^[185]^ and immunotherapy^[186]^ approaches to overcome drug resistance is also being tested in diverse cancers.

## Conclusions and future directions

Treating cancer has become one of the fundamental stumbling blocks of 21st-century medicine. Our efforts to tackle this relentless disease have failed, despite exceptional progress in the field of cancer biology and drug discovery. Hence, there is an urgent need for new therapeutic modalities that can cure cancer. Furthermore, the multi-faceted origin of cancer cells, high mutational rates, and several oncogenic pathways active at a time in these cells emphasize the need for more combination therapies, where the cumulative effect of two or more drugs is much more effective than monotherapy.

On the other hand, one of the prime reasons for cancer drug resistance and relapse is CSCs^[187]^. Multiple studies point out that CSCs are inherently resistant to drug treatment and are known as drug-tolerant persister cells (DTPs)^[187]^. These DTPs express markers like ABCB5, ALDH, CD133, CD271, and KDM5B, which directly or indirectly help in providing resistance against neoplastic drugs. Hence, targeting these persister cells would be one of the ways to effectively target against cancer^[187]^. Also, the role of miRNAs in drug resistance is well established now, and targeting them would be another way to overcome drug resistance. With advances in genomics, proteomics, and metabolomics, it is now feasible to determine the exact molecular signature of individual cancer patients, and personalized medications should be considered for cancer therapy in the future.

In conclusion, the question remains: Is the glass half empty or half full? Perhaps, what we need is a different glass altogether. We are still in the early stages of understanding cancer drug resistance and how to combat it. There is still much to discover and develop novel anti-MDR drugs to improve the treatment of cancer patients.
